# Label free, electric field mediated ultrasensitive electrochemical point-of-care device for CEA detection

**DOI:** 10.1038/s41598-021-82580-y

**Published:** 2021-02-03

**Authors:** B. Chakraborty, A. Das, N. Mandal, N. Samanta, N. Das, C. Roy Chaudhuri

**Affiliations:** 1 Department of Electronics and Telecommunication Engineering, Indian Institute of Engineering Science and Technology, Shibpur, West Bengal 711103 India; 2 School of Electrical Sciences, Indian Institute of Technology Goa, Ponda, 403401 Goa India; 3 Department of Electronics and Communication Engineering, Techno India University, Sector V, Kolkata, 700091 West Bengal India; 4 Department of Electronics and Communication Engineering, KL University, Green Fields, Vaddeswaram, Andhra Pradesh 522502 India

**Keywords:** Health care, Engineering, Nanoscience and technology

## Abstract

Developing point-of-care (PoC) diagnostic platforms for carcinoembryonic antigen detection is essential. However, thefew implementations of transferring the signal amplification strategies in electrochemical sensing on paper-based platforms are not satisfactory in terms of detection limit (LOD). In the quest for pushing down LOD, majority of the research has been targeted towards development of improved nanostructured substrates for entrapping more analyte molecules and augmenting the electron transfer rate to the working electrode. But, such approaches have reached saturation. This paper focuses on enhancing the mass transport of the analyte towards the sensor surface through the application of an electric field, in graphene-ZnO nanorods heterostructure. These hybrid nanostructures have been deposited on flexible polyethylene terephthalate substrates with screen printed electrodes for PoC application. The ZnO nanorods have been functionalized with aptamers and the working sensor has been integrated with smartphone interfaced indigenously developed low cost potentiostat. The performance of the system, requiring only 50 µl analyte has been evaluated using electrochemical impedance spectroscopy and validated against commercially available ELISA kit. Limit of detection of 1 fg/ml in human serum with 6.5% coefficient of variation has been demonstrated, which is more than three orders of magnitude lower than the existing attempts on PoC device.

## Introduction

Tumor markers have high practical importance in tumor screening and early diagnosis. In this aspect, carcinoembryonic antigen (CEA), an acidic glycoprotein involved in cell adhesion and expressed during human fetal development is a marker for rectal and other cancers like breast cancer, ovarian cancer, lung cancer and pancreatic cancer^1,2^. CEA is often presented at quite low levels (0–2.5 ng/ml), even in a healthy person. However, during the formation of early tumors, the level of this biomarker in blood starts increasing slowly. Thus, detection of CEA at ultralow concentration will help in monitoring the gradual elevation of CEA level, thereby indicating the progress of tumors which has profound impact on early stage diagnosis, condition monitoring and therapeutic evaluation of diseases^3–5^. Most of the commercially available CEA diagnosis procedures are based on enzyme-linked immunosorbent assay (ELISA), electrochemiluminescence and chemiluminescence enzyme immunoassay.There are also reported practices for CEA detection based on radioimmunoassay, fluorescence assay and others. Though these assays have good selectivity, they mostly require specific markers or labels like enzymes, fluorescent molecules and radioactive elements. Additionally, the need of complex and expensive instruments along with professional operating limit their wide application^6,7^. In this regard, the development of affordable, sensitive and portable biosensors requiring low volume samples, less reagents and enabling rapid detection are of significant interest for disease prognosis and point-of-care testing (POCT) applications.

Towards this direction, electrochemical biosensors have the greatest potential for transformation to POCT devices since low-cost screen-printed electrodes can be integrated with simple electronic devices for rapid detections in a compact and user-friendly handheld system^8–10^. However, the main problem limiting the development of electrochemical sensor is the detection limit which is much higher than the polymerase chain reaction (PCR) or fluorescence measurement^11^. To overcome this challenge, various signal amplification strategies are deployed involving labels, multiple enzymes and nanoparticles to achieve femtomolar detection limits of CEA in serum^12–15^. But, these reagents and enzymes suffer from stability issues, have reproducibility concerns, requires several washing steps and therefore these sensors are still in its infancy to be deployed in point-of-care situations in spite of their excellent detection limits. There are few efforts for realizing such signal amplification strategies on paper-based platforms with a view to translate them for PoC applications but the lowest limit of CEA detection achieved is only in few picograms/ml^13,16–18^.

Applying the most accepted receptor-ligand binding mechanisms, various nanomaterials like graphene, carbon quantum dots, metal oxides and others have been deployed for efficient, label free electrochemical biosensing^19–22^. In order to reduce the detection limit, efforts have been directed towards the development of superior nanostructured platforms for capturing more analyte molecules and elevating the rate of electron transfer to the working electrode. However, thesestrategies have reached saturation. Therefore, in this work, focus has been given on the mass transport augmentation of the analyte towards the sensor surface with the utilization of an electric field. As a biosensing electrode, graphene-ZnO nanorods hybrid structure has displayed good electrocatalytic activity and high electron transfer rates, due to the synergistic effect and increase in surface area^23,24^. Further, ZnO has high isoelectric point which makes it suitable for adsorption of biomolecules. Moreover, for non-enzymatic label free immunosensing of CEA using electrochemical biosensors, ZnO nanorods can have additional advantage of effectively bridging the gap between them through capture of just one target molecule, thereby amplifying the reduction in exposed sensor area for electron transfer and producing detectable changes in measurable signal for trace concentration of CEA. To the best of our knowledge, graphene-ZnO nanorod heterostructures have not been explored for electric field mediated, electrochemical non-enzymatic protein detection.

Recently flexible substrate-based sensors have emerged as key components of field deployable and wearable devices for POCT applications. Flexible strips also allow low volumes of fluid absorption within the pores, ensuring more effective conjugation and improved sensitivity in the detection of target analyte. Graphene field effect transistor sensors and ZnO nanostructured based electrochemical sensors on flexible substrates have been demonstrated^25,26^. However, these devices have used either lithographically patterned electrodes or solid electrodes which increase the cost and reduce the portability aspects. On the other hand, screen printed electrodes offer the benefits of low cost, easy manufacturability and mass production capabilities. There are few reports of fabrication of screen-printed electrodes on flexible PET substrates^27,28^. But there are almost no efforts of developing graphene-ZnO nanorod heterostructure on flexible substrates with screen printed electrodes (SPEs). Usually commercially available screen-printed electrodes are modified with reduced graphene oxide by drop casting method. This leads to non-uniformity in thickness of the thin film and deposition at a specific location is challenging which eventually results in large sensor to sensor variation^29^. Further growth of vertically oriented ZnO nanorods will be affected as it is dependent on the uniformity of the underlying graphene layer^23^. In this aspect, dielectrophoresis (DEP) is a recommended method since it allows localized controlled deposition, has the ability to be performed at room temperature, requires short time of deposition and has appreciable controllability and uniformity^30^.

In this study, we have re-designed the working electrode for DEP deposition of graphene, followed by ZnO nanorod development on flexible PETsubstrate for electrochemical CEA detection. Thedistinguishable features of this aptasensor with respect to the existing ones includeelectric field mediated label free electrochemical detectioncoupled with the deployment of graphene/ZnO nanorod working electrode on screen printed PET substrate. This unique combination has pushed down the detection limit bymore than three orders of magnitude in comparison to recently reported labeled ZnO nanoparticle-RGO electrochemical sensor^3^. The ZnO nanorods have been immobilized with DNA aptamers against CEA using silanization protocol. Aptamers have the advantages of specificity and stability, making them attractive for POCT applications. To achieve high sensitivity for low CEA concentrations, external electric pulses have been applied with respect to the reference electrode which have been optimized to a value of + 0.8 V. Additionally, a smartphone based handheld potentiostat has been developed which can apply the pulse during the capture phase, perform electrochemical impedance spectroscopy and extract information about target concentration from pre-calibrated data, thereby enabling compact and reliable readout which is a critical element for field sensing. To summarize, the present electrochemical aptasensor holds the advantages of affordability, convenient operability, mass production capability and portability along with significantly low detection limit compared to existing POCT devices for CEA.

## Results and discussions

### Characterization of SPEs and Graphene-ZnO nanorod hybrid

The square resistances of SPEs have been measured to vary between 90 and 95 Ω/□ which is quite satisfactory, as evidenced from a typical electrochemical measurement (Figure S1). The electrochemical response of the SPEs show peak oxidation current around 450 μA and peak potential difference of about 105 mV, which is in fact improved from that of commercially available DropSens electrodes. The SEM image of the working electrode (Figure S2) shows that there is certain amount of surface roughness. It has been observed from the top and cross-sectional SEM images that the ZnO nanorod arrays have been uniformly developedwith sufficient packing density on graphene (Figure S3). The thickness of the graphene layer has been estimated to be around 10 nm from surface profilometry studies. In surface profilometry, a diamond stylus is first shifted vertically in contact with the sample and then laterally across the sample for a specified distance. The profilometer estimates small surface variations in vertical stylus displacement as a function of position which is an indication of thickness variation in the thin film with lateral distance. The Dektak 150 stylus profilometer which has been used to estimate the graphene surface roughness has a12.5 μm radius diamond tip loaded with a force of 0.3 mN (which minimizes scratching of the surface). The scan duration has been set such that the traversing resolution is better than 0.05 μm and the vertical resolution is less than 0.002 μm. From the XRD patterns of graphene, a large reflection peak centered around 2*θ* = 25° has been observed, which can be attributed to an interlayer spacing of approximately 0.36 nm. For the ZnO/graphene heterostructure, the diffraction peaks have been observed in the range of 30° < 2*θ* < 60° which can be assigned to the hexagonal wurtzite structure of ZnO with lattice constants *a* = 0.325 nm and *c* = 0.521 nm. These agree well with previously reported literature^31^. The spectrum from thephotoluminescence spectroscopy (PL)shows a strong peak at 382 nm, which is in good agreement with the band edge emission of ZnO (Figure S5).The Fourier transform infrared (FTIR) spectrum of the ZnO-nanorods/graphene hybrid nanostructure recorded after aptamer functionalization using IR Affinity-1, SHIMADZU, spectrophotometer in kBr medium in the mid IR range of 500–4000 cm^−1^ (Figure S6), exhibits bands around 2800 cm^−1^ and 2900 cm^−1^.This may be attributed to the symmetric and asymmetric stretching of C-H bonds of graphene. There are peaks around 1600 cm^−1^ and 3350 cm^−1^ generated from C = C and OH bonds respectively. Symmetric and asymmetric CQO stretching modes of ZnO results in the bands at 1585 cm^−1^ and 1412 cm^−1^ respectively. The covalent attachment of aminosilane to the OH group of ZnO is validated by the presence of the characteristic peaks at 1100 cm^−1^ and 1005 cm^−1^ corresponding to the symmetrical stretching vibrations of Zn–O–Si and Si–O–Si, respectively. Cyclic voltammetry (CV) studies have been performed to explore the electrocatalytic behavior of the fabricated electrodes towards the redox electrolyte [K_4_Fe(CN)_6_]. CV of fabricated SPE, both before and after aptamer binding conditions, shows that the peak oxidation current has been reduced to 250 μA from 350 after aptamer binding (Figure S7).

### Electrochemical response and optimization of electric field

**Figure 1:**
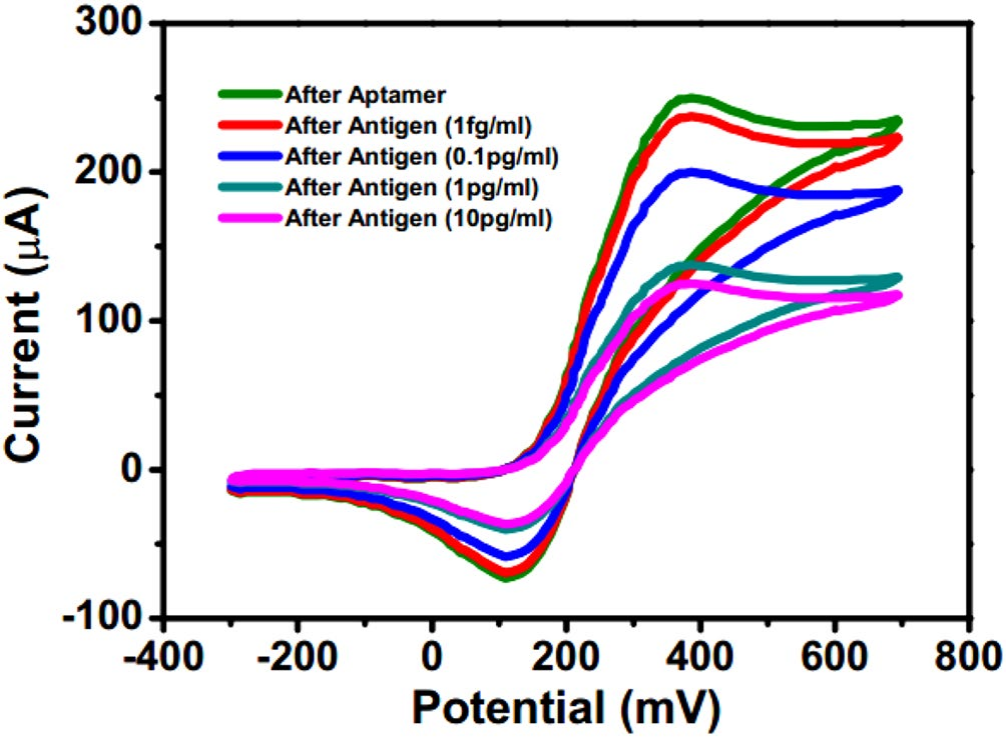
Cyclic voltammetry in absence of external electric field after aptamer and antigen binding (graph indicates mean values with maximum standard deviations of ± 5%).

**Figure 2:**
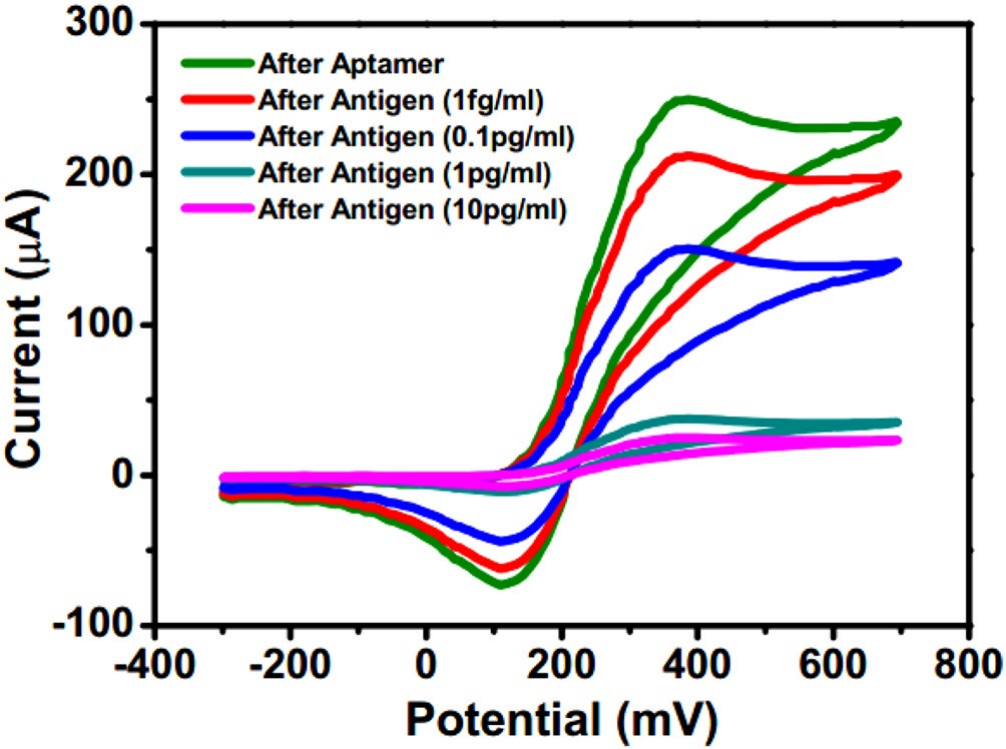
Cyclic voltammetry in presence of external electric field after aptamer and antigen binding (graph indicates mean values with maximum standard deviations of ± 6%).

**Figure 3:**
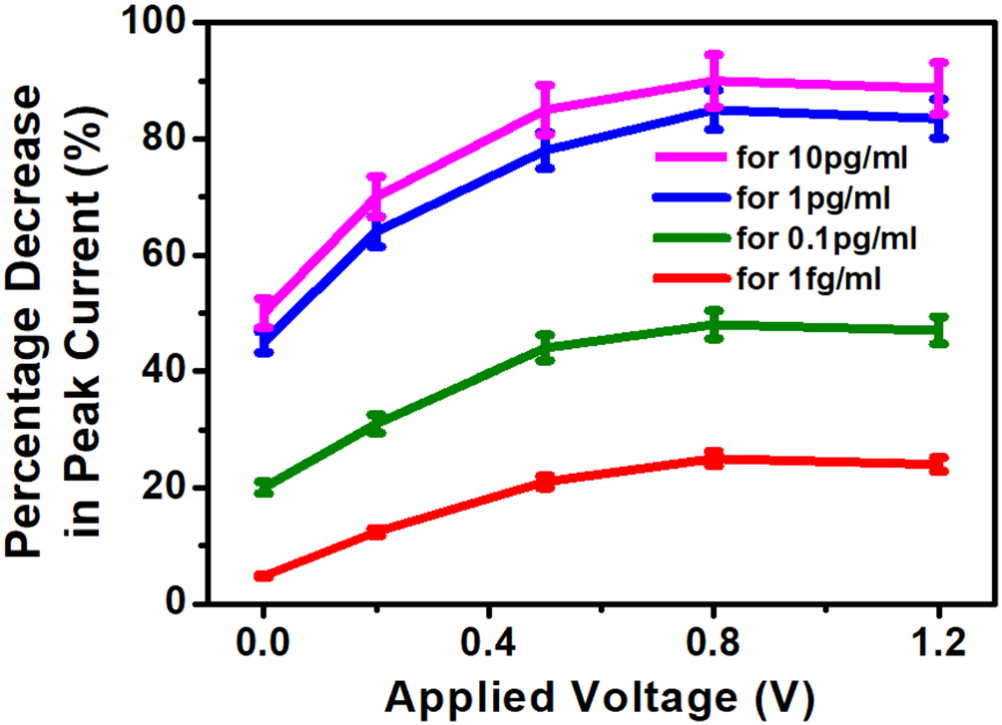
Percentage decrease in peak current at differ ent applied voltages.

To investigate the change in the electrochemical response of the sensor before and after loading of CEA, CV measurements have been conducted initially in the absence of any additional electric field. It can be observed from Fig. 1 that the decrease in the peak oxidation current is quite satisfactory for CEA in the concentration of 100 fg/ml to 10 pg/ml which may be attributed to the hindrance in the flow of electrons from solution to electrode through the ZnO-graphene heterostructure. But for lower concentration, the decrease is not substantial and cannot be differentiated from the deviations in the baselinecurve. To achieve more sensitive detection, an external pulse has been applied during CEA loading time. However, the pulse intensity needs to be optimized since with higher magnitude, electroporation of antigen might be facilitated^32^. To determine the optimal condition for CEA sensing, the dependence of the loading efficiency on pulse intensities of 0, + 0.5, + 0.8 V and + 1.2 V have been investigated through CV measurements. Figure 2 shows a typical CV plot before and after CEA capture in presence of pulse intensity of + 0.8 V which indicates significant decrease in peak current even at 1 fg/ml. The percentage decrease in peak current for different pulse intensities and CEA concentrations has been represented in Fig. 3 which indicates that at both + 0.8 and + 1.2 V, it has greatly enhanced in the low analyte concentration rangecompared to that without pulsed conditions. This may be attributed to the increased possibility of more directed capture upon application of perpendicular electric field. However, with the higher pulse of + 1.2 V, the range of measurement gets limited to 1 pg/ml.

**Figure 4:**
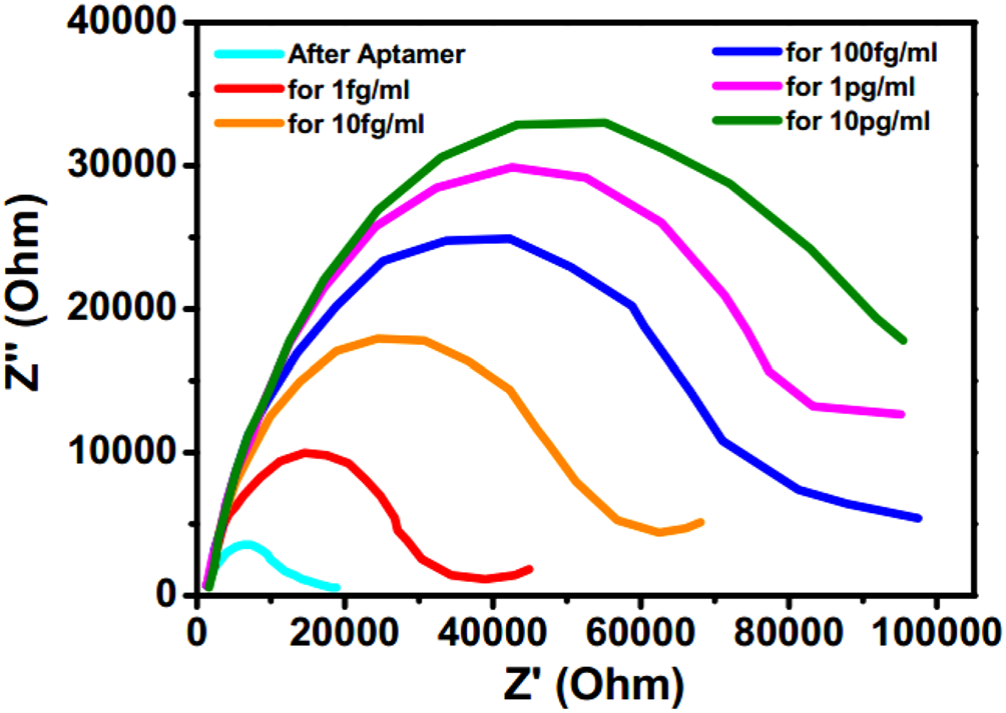
EIS of Graphene ZnO sensor electrode before and after capture of 1 fg/ml to 10 pg/ml of CEA with external pulses of + 0.8 V. (graph indicates mean values with maximum standard deviations of ± 5.6%).

**Figure 5:**
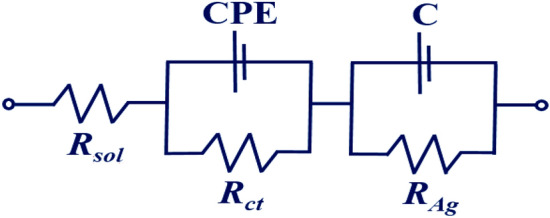
Equivalent circuit diagram.

**Figure 6:**
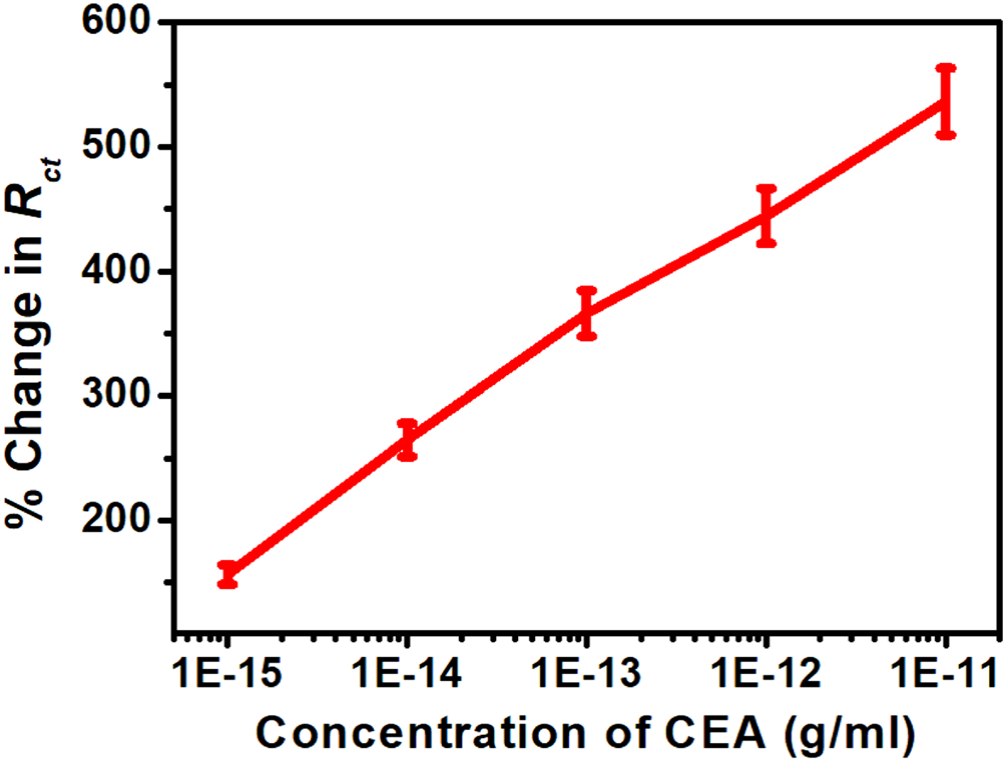
Rct versus CEA concentration.

**Figure 7:**
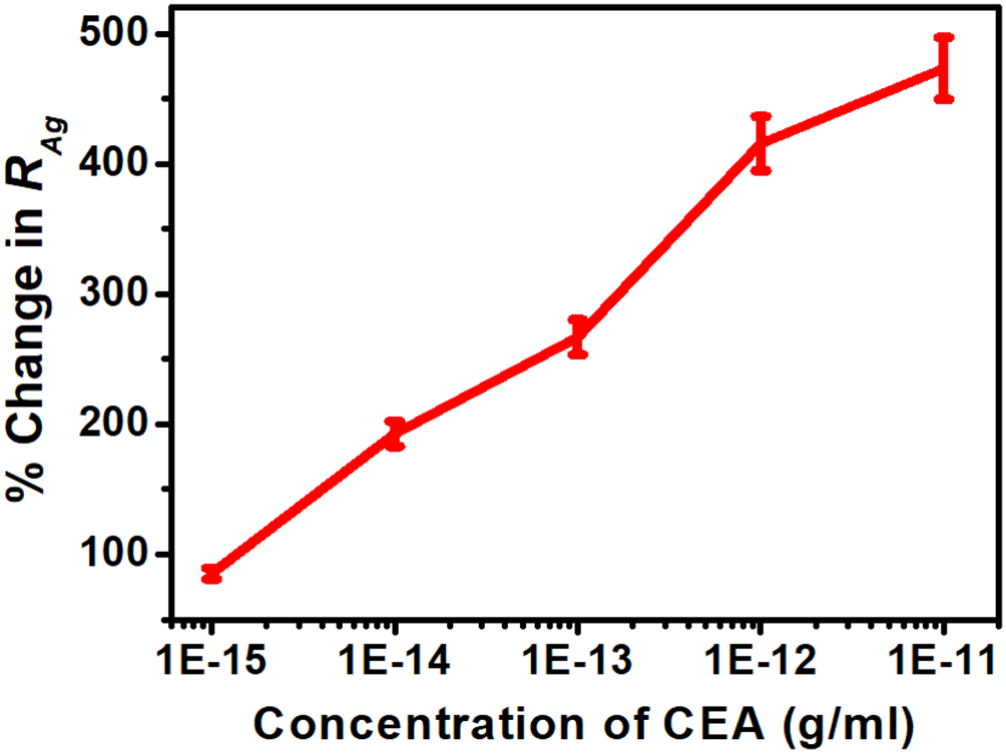
RAg versus CEA concentration.

**Figure 8:**
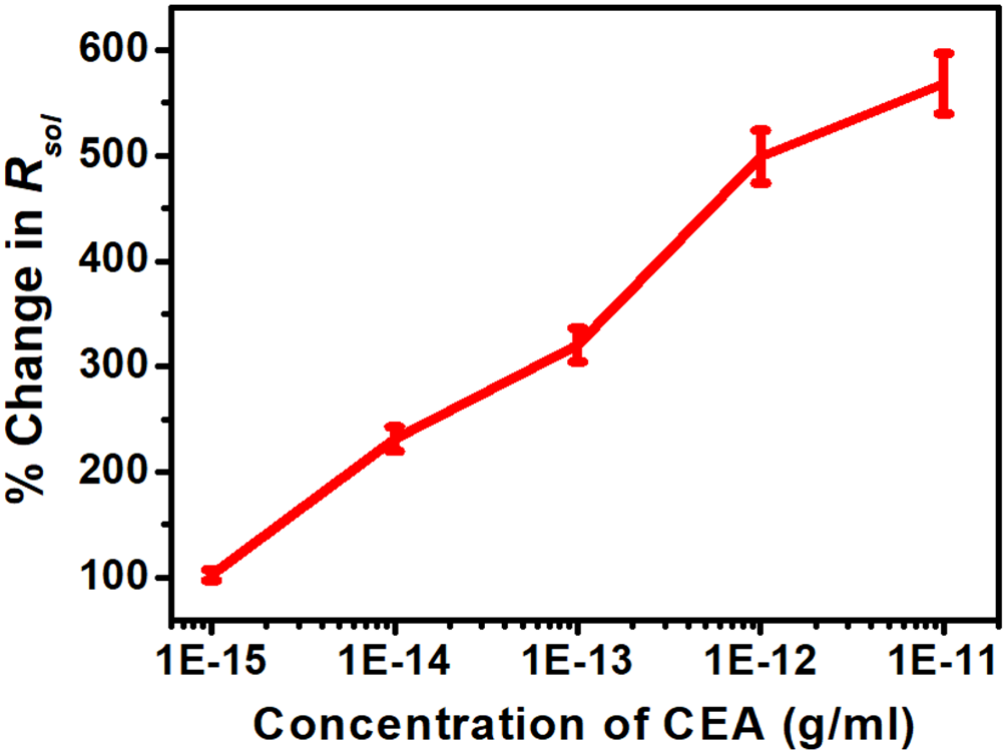
Rsol versus CEA concentration.

To examine the individual contributions within the heterostructure, Nyquist plots have been generated before and after application of CEA for an applied electric field of + 0.8 V in the frequency range of 1 Hz to 100 kHz and have been fitted with an equivalent circuit depicted in Figs. 4 and 5 respectively. The response of the sensor has been observed to be steady within 8 min. In the plot, Z^/^ and Z^//^ represents the real and imaginary component of the impedance respectively. The model comprises of a bulk solution resistance (*R*_*sol*_), double layer capacitance (*C*) formed by the ions at the vicinity of the electrode, the charge transfer resistance (*R*_*ct*_) that represents the current flow due to redox reactions at the electrode–electrolyte interface, a constant phase element (CPE) and the possible resistance of the attached CEA molecules with the ZnO (*R*_*Ag*_). Interestingly, the Nyquist graphs and fitted values in Figs. 6, 7 and 8 show that the most significant changes occur not only in *R*_*ct*_ and *R*_*Ag*_ values but also in *R*_*sol*_ which may be attributed to the significant bridging of gap between the ZnO nanorods after capture of CEA. These phenomena accompanied by the application of electric pulse during antigen loading have enabled to lower the detection limit to 1 fg/ml.

### Selectivity assessment of the sensor

As the immobilized aptamers control the main interaction between the analyte molecule and the sensor electrode, it is expected that the biosensor should possess sufficient specificity. To investigate the selectivity of the biosensor, the sensor electrode has been tested with other antigen like thrombin, BSA and PSA at concentrations of 1 ng/ml. It has been observed from the Nyquist plot (Figure S8) that the biosensor responses to other molecules are significantly lower, which makes the sensor specific for the target analyte.

### Detection of CEA in serum

**Figure 9:**
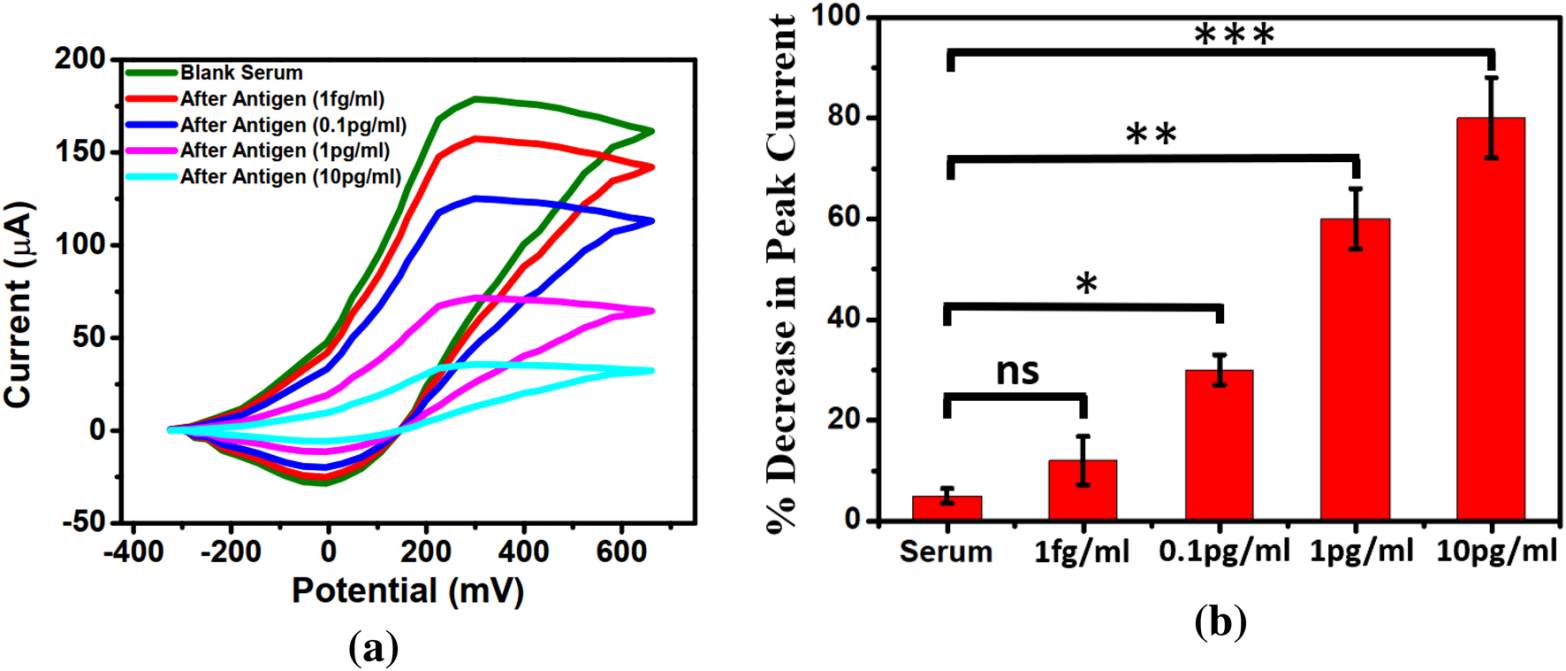
(**a**) CV in presence ofserum. (**b**) Percentage decrease in peak current with different CEA concentration in serum (ANOVA). Error bars represent the standard deviation (ns: *p* > 0.05 and *p < 0.01, **p < 0.001, ***p < 0.0001).

To validate the practicability of the proposed sensing methodology, detection of different concentrations of CEA in serumhas been investigated. For this purpose, both the CV andNyquist plots have been generated first in blank 50% serum and then with CEA spiked serum. From the CV plot (Fig. 9a), it has been observed that in presence of serum, the shape is somewhat distorted and the sharpness of the redox peaks is also reduced. This may be ascribed to the interferents in serum which increases the double layer capacitance and decreases the ratio of faradaic to capacitive charging current. The mean and standard deviations of the percentage decrease in current peak has been estimated for different concentrations of CEA and represented in Fig. 9b. It can be observed that though the mean percentage change in current for 1 fg/ml is around 12%, there is a significant standard deviation which reduces the difference with blank serum. This may be attributed to the randomness in the non-Faradaic component of charging current, resulting from interferents, which increases the noise in the measurement of peak current. However, for higher concentrations of CEA, as the decrease in peak current is substantial, this problem reduces. Hence from CV plot, the LOD achieved is 100 fg/ml.

**Figure 10:**
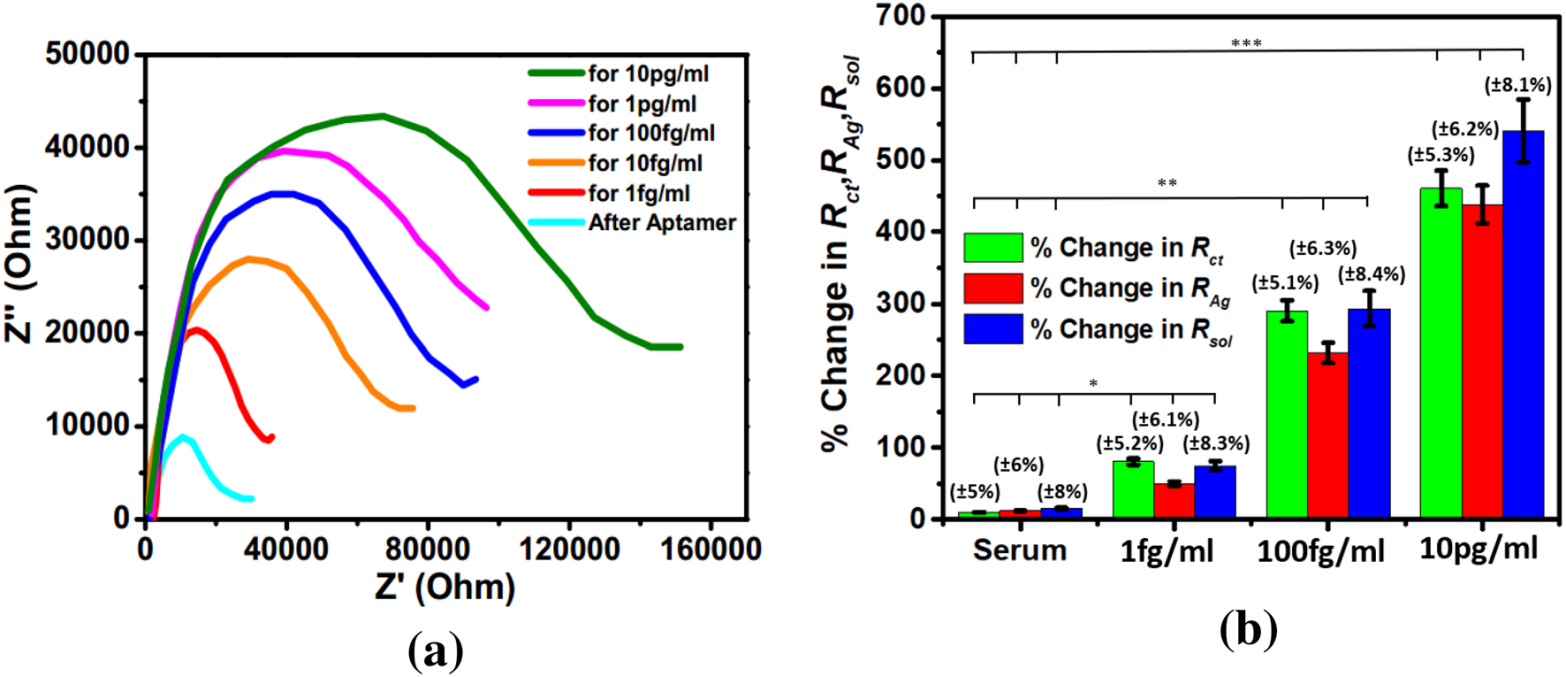
(**a**) EIS of Graphene-ZnO sensor electrode before and after capture of 1 fg/ml to 10 pg/ml of CEA in serum with external pulses of + 0.8 V. (graph indicates mean values with maximum standard deviations of ± 6.2%). (**b**) Percentage change in Rct, Rsol and RAg with different CEA concentration in serum. Error bars represent the standard deviation. Statistical analysis (ANOVA) shows significant difference with blank serum. (*p < 0.01, **p < 0.001, ***p < 0.0001).

In the Nyquist plot, the relatively large impedance of approximately around 8300 Ω of bare electrode in blank serum may be ascribed to the non-specific interaction of various interfering proteins in the matrix (Fig. 10a). Additionally, the interferents in serum causing greater double layer capacitance on the surface of the sensor electrode results in slightly different patterns of all impedimetric curves from their previously measured buffer experiments. Due to the presence of high amount of interferences, the average change in *R*_*ct*,_ *R*_*Ag*_ and *R*_*sol*_ are 80%, 50% and 75% respectively for 1 fg/ml CEA which are lower than that in buffer. For statistical analysis, the mean and standard deviations of the percentage changes in *R*_*ct*_, *R*_*Ag*_ and *R*_*sol*_ have been estimated for three representative samples in the lower, middle and higher concentration range of CEA, as shown in Fig. 10b. It has been observed that there is a considerable difference in all the three parameters between the blank medium which is unspiked human serum and each of the CEA concentration. Hence, clearly the LOD demonstrated is 1 fg/ml in human serum. The analytical parameters of the aptasensor have been compared with the already reported biosensors. Summarization of some of the attributes of the fabricated electrode and other sensors has been represented in Table 1 which clearly indicates that our proposed sensor has a lower detection limit than most of the labeled CEA electrochemical sensors.

**Table 1:** A comparison of some previous biosensors and present biosensor for CEA detection.

Electrode materials	Linear range	LOD	Ref
ZnO flower-rods modified with g-C3N4-Au nanoparticle nanohybrids	0.01–2.5 ng/ml	1.9 pg/ml	^6^
Hollow porous gold nanoparticles	2–100 pg/ml	1.5 pg/ml	^7^
Aptamer functionalized Au electrode	3 pg**/**ml to 40 ng**/**ml	0.9 pg**/**ml	^14^
DNA tetrahedron probes decorated Au electrode	0.0001–50 ng/ml	18.2 fg/ml	^15^
Hemin, graphene oxide and multi-walled carbon nanotubes modified glassy carbon electrode	1.0 × 10^–15^–1.0 × 10^−8^ g/ml	0.82 fg/ml	^33^
ZnO-RGO composite modified carbon paste electrode	0.01–6.0 ng/ml	4.0 pg/ml	^3^
Au nanoparticle decorated graphene modified glassy carbon electrode	0.1–1000 ng/ml	60 pg/ml	^34^
PdAuPt nanoparticles decorated RGO modified gold electrode	0.012–85 ng**/**ml	8.0 pg**/**ml	^35^
Au nanoparticle decorated RGO modified glassy carbon electrode	0.05–0.65 ng**/**ml	5.3 pg**/**ml	^36^
Graphene/ZnO nanorod modified flexible PET substrate	0.001–10 pg/ml	1 fg/ml	This work

### Calibration of smartphone readout

**Figure 11:**
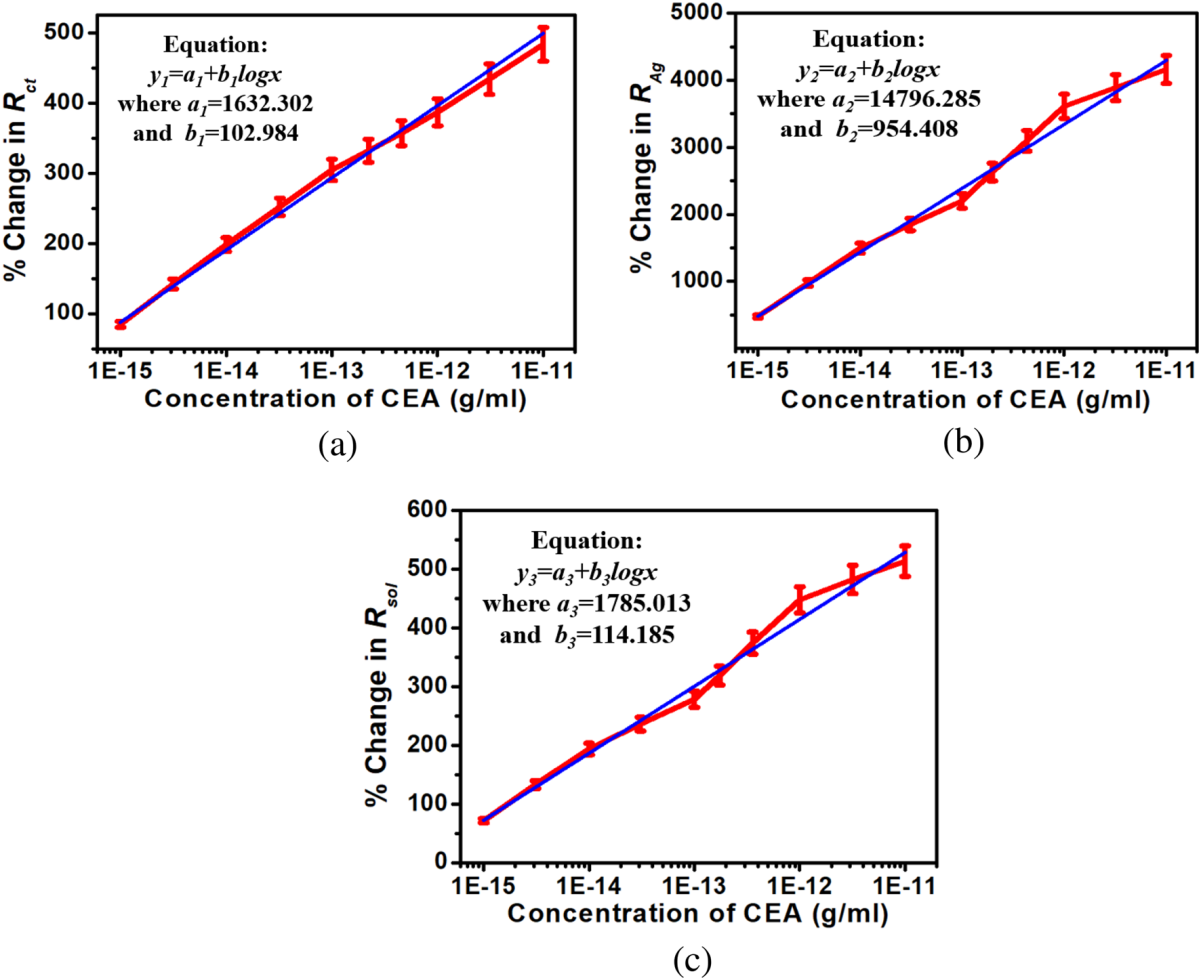
Calibration curves (**a**) Rct, (**b**) RAg and (**c**) Rsol with CEA concentration.

For the purpose of calibration, the handheld potentiostat system measures the Nyquist plot and processes the data to extract *R*_*ct*_, *R*_*Ag*_ and *R*_*sol*_ for serum as control and ten different CEA concentrations spiked in serum. The corresponding percentage changes are transferred to the smartphone by enabling the Bluetooth module via UART port serially. The smartphone app stores these data for five sensors against a particular CEA concentration, generates the mean and standard deviations, plots the graphs and computes an empirical relationship (given in Eq. 1) between CEA concentration and percentage changes in *R*_*ct*_*, R*_*Ag*_ and *R*_*sol*_ as shown in Fig. 11.

1 $$ \log x = - 15.661 + \frac{1}{3}\left( {\frac{{y_{1} }}{102.984} + \frac{{y_{2} }}{954.408} + \frac{{y_{3} }}{114.185}} \right) $$

where *x* is the concentration of CEA; *y*_1_, *y*_2_ and *y*_3_ are percentage changes in *R*_*ct*_, *R*_*Ag*_ and *R*_*sol*_ respectively.

### Development of smartphone app

The smartphone app has two sections-calibration and concentration estimation. Once the app is activated, the user has to select the appropriate function. In the calibration mode, the app generates the empirical relationship between CEA concentration and percentage changes in *R*_*ct*_*, R*_*Ag*_ and *R*_*sol*_ as discussed in previous section. In concentration estimation mode, the app accepts the percentage changes in *R*_*ct*_*, R*_*Ag*_ and *R*_*sol*_ from the potentiostat for an unknown CEA solution, computes the concentration of CEA based on the embedded calibration function, repeats the process for five sensors, estimates the mean concentration along with its standard deviation and finally displays it. This app (which is .apk file) has been designed to be compatible with Android OS 2.3 (API level 9) and higher Android versions. The app, when installed into the android system requires permissions like accessing Bluetooth settings and pairing permission with other bluetooth devices so that the smartphone can pair up with the potentiostat for data analysis. All the data transmitted to the smartphone has been designed to be stored in a default folder in the internal storage of the smartphone, hence the app requires permission to read, modify or delete contents of internal storage. It also needs permission for viewing and establishing Wi-Fi connections with full network access to send data to stakeholders. The android interface has been incorporated with a special “send button”. If this button is clicked, then the evaluated CEA concentration can be sent to the person concerned by activating the WiFi of the smartphone.

For testing the system, the unknown solution has been pippetted onto the screen-printed sensor, interfaced with the potentiostat which then allows for the capture of CEA under the influence of electric field and computes percentage changes in *R*_*ct*_, *R*_*Ag*_ and *R*_*sol*_. The smartphone app then acceptsthese data from the potentiostat and finally displays the mean concentration along with the standard deviation, as discussed above. The CEA concentration extracted from the sensing system has been compared with that obtained from commercially available human CEA ELISA kit (Biorbyt). For validating test samples with CEA concentration lower than the detection limit of the ELISA kit, the measurements obtained from the commercial system have been divided by the dilution factor which has been used for preparing the corresponding low concentration CEA samples. This reading has been correlated with that obtained from the sensing system. Table 2 indicates the mean concentration along with its coefficient of variation (%CoV) obtained from the proposed PoC device and the ELISA kit. It is observed that the readings obtained from the sensor system agree well with that of the gold standard. Hence, this mobile app will enable CEA quantification and online transmission of data, if required. A comparison of the proposed PoC system with the existing reports on PoC device for CEA detection is represented in Table 3 which indicates that the detection limit is more than three orders of magnitude lower.

**Table 2:** Validation of the proposed sensor.

Serum samples with CEA (concentration)	Results			
	Using proposed sensor		Using Commercial ELISA kit	
	Mean	%CoV	Mean	%CoV
2 fg/ml	2.3 fg/ml	± 5%	2.20 fg/ml	± 4.8%
5 pg/ml	5.2 pg/ml	± 1.5%	5.1 pg/ml	± 1.4%
10 pg/ml	10.4 pg/ml	± 0.8%	10.1 pg/ml	± 0.6%
300 fg/ml	305 fg/ml	± 3.5%	302.1 fg/ml	± 3.4%
1 pg/ml	1.2 pg/ml	± 2.3%	1.1 pg/ml	± 2.1%
20 fg/ml	21 fg/ml	± 4.4%	22 fg/ml	± 4.1%

**Table 3:** A comparison of the proposed PoC system with existing reports.

Reference	Detection limit	Range
^18^	6.7 pg/ml	0.05–20 ng/ml
^13^	0.15 ng/ml	0.50–70 ng/ml
^17^	3 pg/ml	0.01–20.0 ng/ml
^16^	5 ng/ml	–
This paper	1 fg/ml	0.001–10 pg/ml

## Conclusion

In this paper, a PoC system for CEA detection has been demonstrated based on label free, electric field mediated electrochemical detection using graphene-ZnO nanorod heterostructure with screen printed electrodes on PET substrate and smartphone interface. It has been observed that at an optimum electric field corresponding to 0.8 V with respect to the reference electrode, a four times enhancement occurs in the sensor response. Further the Nyquist plot shows that there is appreciable change in the solution resistance upon antigen capture unlike most of the working electrodes, which may be attributed to the significant bridging of gap between the ZnO nanorods after capture of CEA. This approach has enabled to achieve a detection limit of 1 fg/ml, which is more than three orders of magnitude lower than the existing PoC devices. To summarize, the proposed electrochemical aptasensor with the advantages of high sensitivity, affordability, convenient operability, mass productioncapability and portability can be extended for multiple target analysis, thereby introducing them as new potential PoC devices for various biomarker detection.

## Methods

### Materials and reagents

An aptamer has been selected with a sequence of: 5′-NH2 ATACCAGCTTATTCAATT-5′ as indicated by other researchers^33^. Then1 μM aptamer solution has been processed with a TE buffer (10 mM Tris-HCl, 1 mM EDTA, 0.1 M NaCl, pH = 7.40) and stored prior to use at − 20 °C^33^. Carbon ink (No.CH-8) has been procured from Jujo Chemical Co. Ltd., Japan. Silver conductive ink (No. BY-2100) has been purchased from Shanghai Baoyin Electronic Materials Ltd., China. PET films (100 μm) have been procured from Baoding Lucky Innovative Materials Co. Ltd., China. Zinc acetate dehydrate (98%), graphite powder, zinc nitrate hexahydrate (Zn(NO_3_)_2_, 6H_2_O) (98%), hexamethylenetetramine (C_6_H_12_N_4_) (99%), sodium hydroxide, 4-Maleimidobutyric acid N-hydroxysuccinimide ester (GMBS) and 3-mercaptopropyltrimethoxysilane (MTS) have been bought from Sigma-Aldrich. Sodium nitrate (NaNO_3_), sulphuric acid (H_2_SO_4_), potassium permanganate (KMnO_4_), hydrogen peroxide (H_2_O_2_), hydrochloric acid (HCl) and ammonium hydroxide (NH_4_OH) solutions have been procured from Merck.

### Fabrication of SPEs on PET substrates

**Figure 12:**
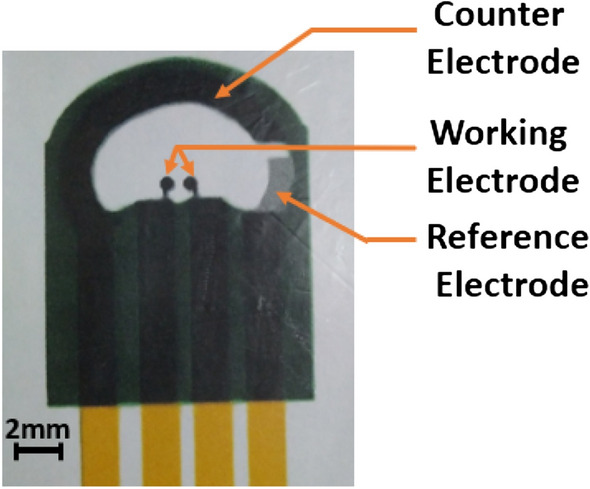
The schematic representation of the SPE.

For precise and quality printing, controlling surface affinity and thermal stability of PET substrates are crucial factors. After cleaning the PET substrates, they have been heated at 120 °C for 15 min. As ink adhesion is influenced by the hydrophilicity of the substrate, the contact angle (measured using Advanced Goniometer from Rame-Hart Instrument Company) of water on both sides of the film has been detected to be around 55 °C. High precision manual screen printing equipment has been used to fabricate the SPEs on PET substrates. First, silver ink has been printed to act as conductor and reference electrode and then carbon ink has been printed on the silver layer to define the working and counter electrode. After each step, the film has been cured at 120 °C for approximately 1 h. Finally insulating ink has been printed on the carbon layer and heated at 120 °C for 15 min for curing. These SPEs have then been soaked into 3 M sodium hydroxide (NaOH) solution for 1 h as chemical treatment. This has been followed by anodization in 0.5 M NaOH solutions by applying 1.2 V anodic potentials for 20 s as electrochemical treatment. Similar process has been reported by other researchers^27^. The schematic representation of the SPEs is shown in Fig. 12. The sensing electrode, i.e. the working electrode is split in two parts, each of approximately 0.75 mm^2^ area with a gap of 500 μm. During deposition of graphene by DEP method, a differential potential has been applied across them, whereas during electrochemical measurements, they have been shorted externally.

### Preparation of graphene-ZnO nanorod heterostructure

Colloidal graphene solution has been pipetted on the sensing area for graphene deposition by DEP process so that the solution droplet covers the working electrodes. Then a sinusoidal voltage for 60 s has been applied (peak to peak 19.5 V). To improve the adhesion, the graphene deposited SPEs have been heated for 20 min at 75 °C^37^. After that, ZnO seed layer has been grown on graphene deposited SPEs incorporating sol–gel method. For this intention, 50 mM Zn(NO_3_)_2_,6H_2_O has been blended with 100 ml DI water to prepare ZnO precursor solution. On a magnetic stirrer, the solution bath has been retained and heat has been supplied. As soon as the bath temperature rises to 60 °C, 4 ml of NH_4_OH solution has been poured into the solution bath. This has been accompanied by 30 min of stirring. Subsequently, the solution bath has been stored undisturbed for 24 h. Then ZnO film has been spin-coated on graphene-deposited SPEs, obtained from the ZnO precursor solution^38^. Sample has been heated at 70 °C to vaporize the remaining solvent. In the second stage, following earlier reported process, ZnO nanorods have been grown on the seeded area^39^. 50 mM Zn(NO_3_)_2_,6H_2_O has been added in 50 ml DI water and stirred for 10 min. 50 mM C_6_H_12_N_4_ has been added with 50 ml DI water and this has also been stirred for 10 min. Then both the solution baths have been combined together followed by stirring at 260 rpm. The ZnO seeded graphene deposited SPE has been suspended into the solution bath for 2 h. After that, the ZnO seeded graphene deposited SPE has been removed from the solution bath and washed with DI water. The ZnO nanorods have been annealed at 100 °C for 15 min using rapid thermal annealing technique to obtain better crystallization.

### Functionalization of ZnO nanorod with aptamers

Silanization method has been executed followed by crosslinker attachment process to obtain proper aptamer immobilization. Taking 0.4 ml MTS and 9.258 ml ethanol with 0.341 ml DI water, the silane solution has been formed and pipetted on the ZnO/graphene SPE surface. The SPEs have been then exposed in nitrogen environment for 24 h. After that, the additional silane solution has been rejected and ethanol has been used to wash the SPEs. Then 5 min of ethanol ultra-sonication has been conducted, followed by a drying session in the atmosphere of nitrogen. After this, 2 mM GMBS solution has been pipetted onto the ZnO nanorod surface to complete the crosslinker attachment and kept aside for 24 h to interact. The SPEs have been then ultrasonicated in ethanol for approximately 5 min^38^. For covalent binding of the aptamer, the activated ZnO/graphene SPE surface is now suitable.

### Preparation of CEA solution

The target molecule CEA, has been procured from US Biological, USA (1 mg/ml). Serial dilutions of CEA solution have been prepared in PBS of 100 mM ionic strength and pH 7.2 to obtain concentrations ranging from 1 fg/ml to 10 pg/ml. CEA has also been spiked into the blood serum for measurement in physiological analyte. In order to separate the serum at room temperature, the blood samples have been obtained from a microbiological laboratory and preserved for some time, as indicated earlier^38^. For testing with humans serum samples, the necessary ethical clearance has been obtained from the Institutional Ethics Committee. Biosensor selectivity has been tested with thrombin, PSA and BSA molecules by separately spiking 1 ng/mL of each of them into the serum.

### Characterization of electrodes and sensor material

Four probe resistivity measuring unit (SES Instruments, DFP-02) has been used for detecting the square resistance of SPEs. Electrochemical measurement of both SPEs and graphene-ZnO nanorod sensor has been performed using CV measurements (OrigaFlex-OGF500 pack, France) in a solution containing 1 mM K_3_Fe(CN)_6_ and K_4_Fe(CN)_6_ with a scan rate of 100 mV/sec and the performance has been compared with commercially available three electrode system of DropSens (DRP110, Spain). CV is one of the most commonly used electrochemical techniques whose graphical analysis provides the position of the redox peaks which establishes the behavior of a comparatively new electrode towards the electrolyte. Field emission scanning electron microscopy (ZEISS EVO-MA 10) has been used for observation of surface morphology and surface profilometer (Dektak ST3) has been used for observing the variation in thickness of both SPEs and graphene. PL measurement of ZnO nanorods has been performed using MicroPL System. X-Ray diffraction (XRD) characterization technique (RigakuMiniFlex X-ray diffractometer) has been incorporated to realize the crystal structure of the ZnO nanorods and graphene. FTIR spectroscopy of the ZnO-nanorods/graphene hybrid nanostructure has been performed using IR Affinity-1, SHIMADZU, spectrophotometer.

### Electrochemical sensing of CEA

The graphene-ZnO working electrode after aptamer immobilization has been characterized initially by cyclic voltammetry (CV) measurements. Next, CEA solution has been dropped on the aptamer coated electrode and incubated for 10 min at room temperature. The electrodes have then been washed gently with DI water. At this stage optimized external electric pulses have been applied between the counter and working electrode with respect to the reference electrode. For the purpose of optimization, the field has been varied in the range of + 0.1 V to + 2 V. The electric field has been limited to + 2 V since higher values may cause denaturation. After the optimization of electric field and application of the same, CV measurements have been conducted with CEA solution diluted with 100 mM PBS in the concentration range from 1 fg/ml to 10 pg/ml. Further, electrochemical impedance spectroscopy has been performed to extract various parameters of the electrochemical system. Additionally, 50% human serum spiked with different concentration of CEA have also been tested with the sensors. Each measurement has been conducted with five sets of SPEs (n = 5 replicates) and the mean and standard deviation have been recorded. Limit of detection has been estimated as that CEA concentration for which the peak currents in the CV plot is at least 3 times the standard deviation from the mean baseline value. For the statistical analysis, one-way ANOVA has been performed by using GraphPad Prism (GraphPad Software, La Jolla, CA, USA). The p-value of statistical significance has been set at *p* < 0.05.

### Handheld potentiostat with smartphone interface

As desktop potentiostats are not convenient for POCT applications, it is desirable to develop handheld potentiostats with smartphone interface for data display and transmission from rural centers. We have presented a handheld potentiostat system with flexible voltage range upto ± 4 V and maximum current measuring capability of ± 5 mA. The SPEs have been interfaced with a cable connector for dual working electrodes commercially available. User selectable switches have been incorporated in the hardware to select the application of electric pulse, CV and electrochemical impedance spectroscopy protocol, as desired. This reduces the power consumption of Bluetooth enabled smartphone-based control. However, evaluation of sensor response, display of the CEA concentration of unknown samples and its subsequent transmission have been enabled through the smartphone app. The system has been powered by two lithium-ion 18,650 rechargeable cells with an approximate development cost of around $70.

## Supplementary Information

Supplementary Information.
