# Supplementary material for: Label free, electric field mediated ultrasensitive electrochemical point-of-care device for CEA detection
Source: Sci Rep. 2021 Feb 3;11:2962. doi: 10.1038/s41598-021-82580-y (PMC7859218; doi:10.1038/s41598-021-82580-y)
Supplement: Supplementary file 1 — Supplementary Information. [file 41598_2021_82580_MOESM1_ESM.docx]

**SUPPLEMENTARY INFORMATION**

**Label Free, Electric Field Mediated Ultrasensitive Electrochemical Point-of-Care Device for CEA Detection**

B.Chakraborty, A.Das, N.Mondal, N.Samanta, N.Das, C.RoyChaudhuri^*^


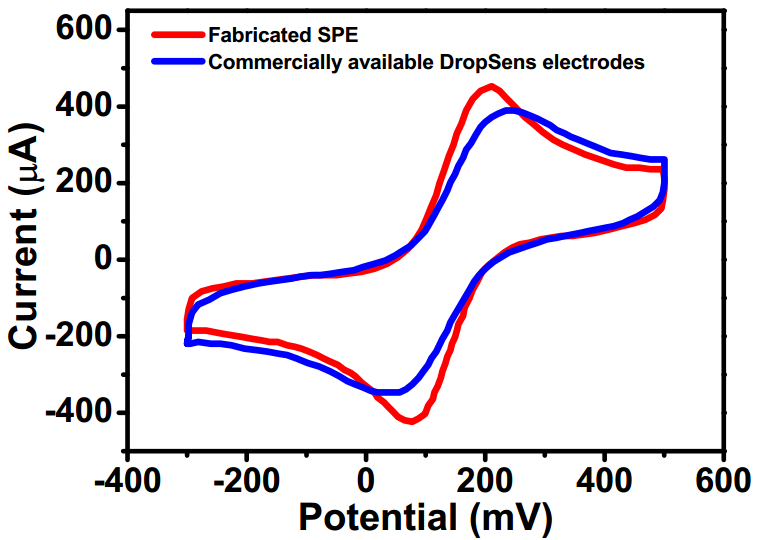


**Figure S1.** Cyclic voltammetry of fabricated SPE and commercially available DropSens electrode.


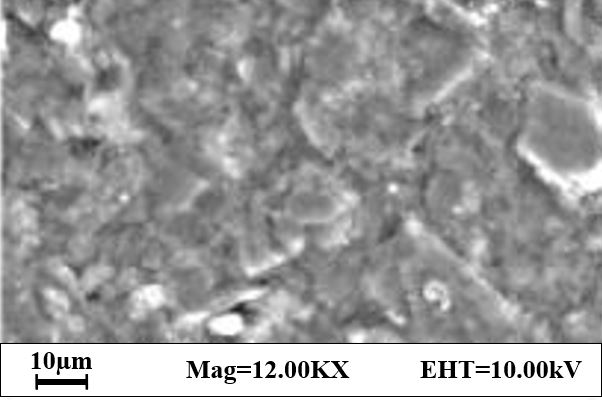


**Figure S2.** SEM image of working electrode surface of SPE.


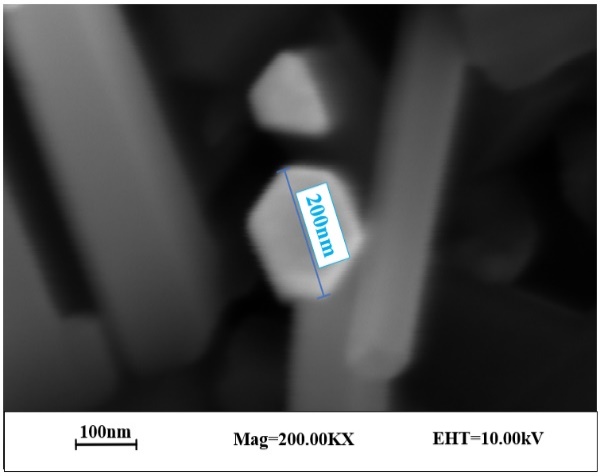

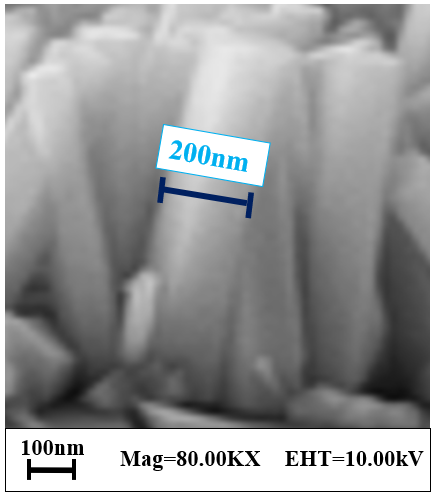


**Figure S3.** SEM images of the Graphene-ZnO nanorod hybrid. (a) Top view SEM image. (b) Cross-sectional SEM image.

**(b)**

**(a)**


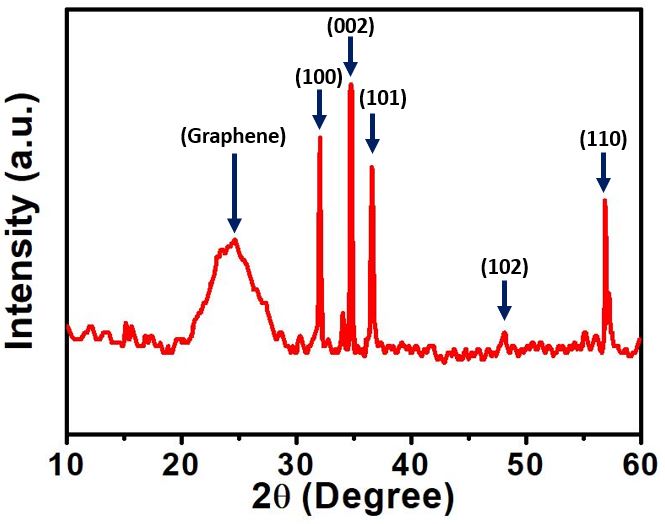


**Figure S4.** XRD pattern of the Graphene-ZnO nanorod hybrid.


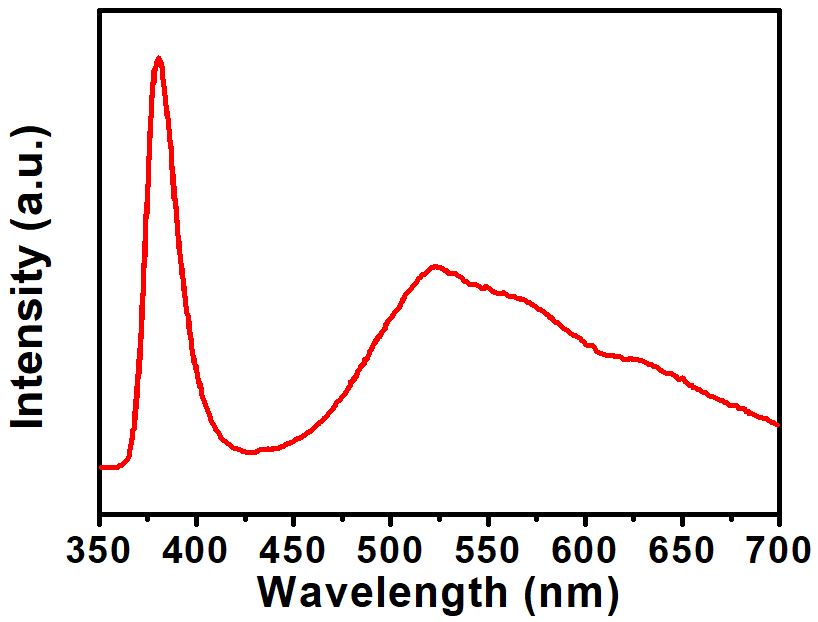


**Figure S5.** Photoluminescence spectrum of the Graphene-ZnO nanorod hybrid.


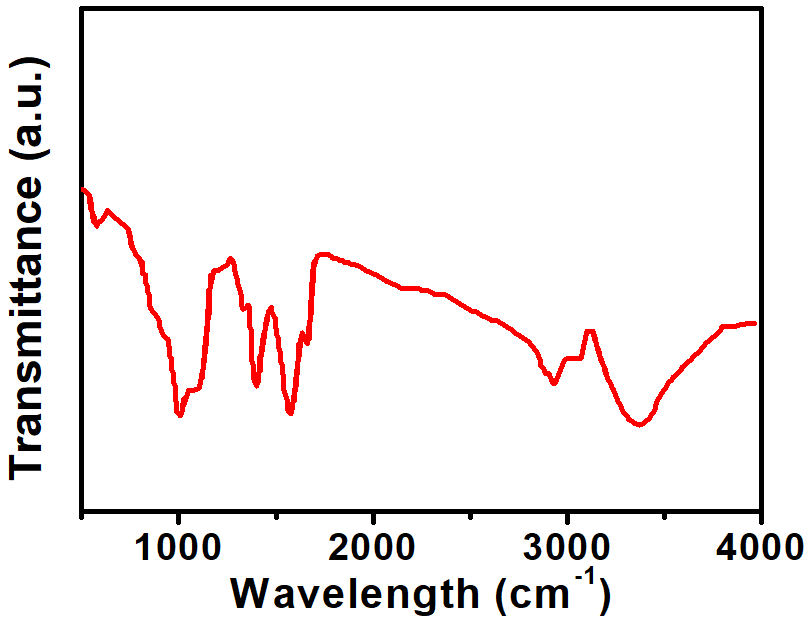


**Figure S6.** FTIR spectrum of the Graphene-ZnO nanorod hybrid.


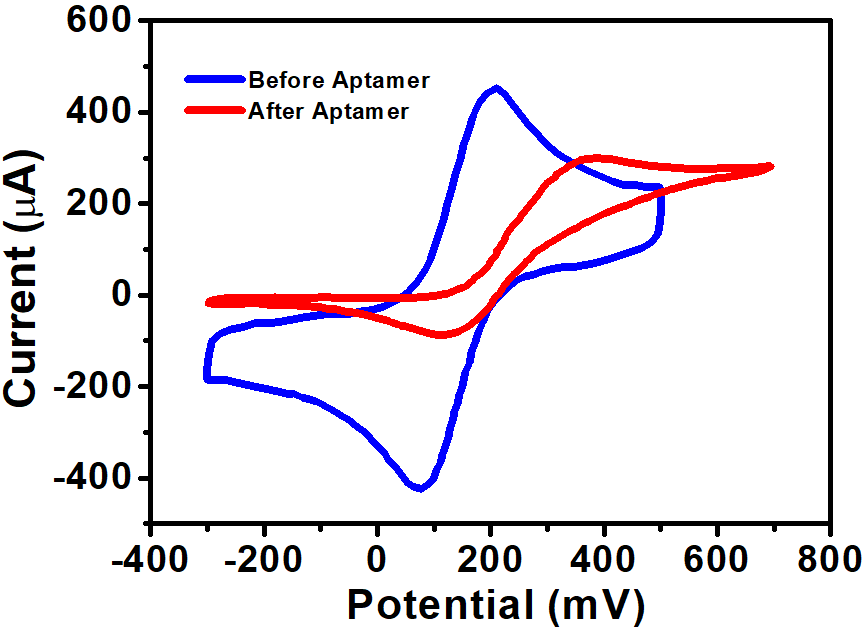


**Figure S7.** Cyclic voltammetry of fabricated SPE before and after aptamer binding.


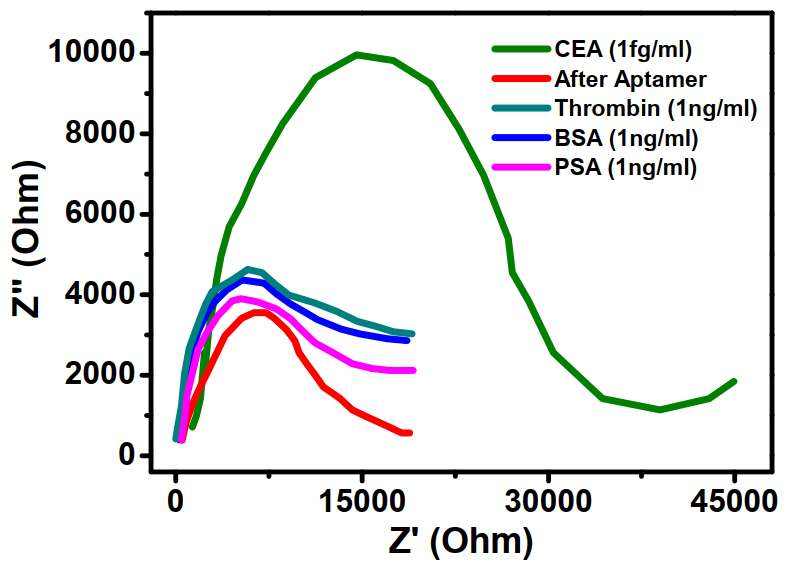


**Figure S8.** Selectivity of the proposed sensor.
